# Wongee Mia: An Innovative Family-Centred Approach to Addressing Aboriginal Housing Needs and Preventing Eviction in Australia

**DOI:** 10.3390/ijerph17155501

**Published:** 2020-07-30

**Authors:** Shannen Vallesi, Eleanor Tighe, Herbert Bropho, Margaret Potangaroa, Leah Watkins

**Affiliations:** 1 School of Population and Global Health, The University of Western Australia, Crawley, WA 6009, Australia; 2 Centre for Social Impact, UWA Business School, The University of Western Australia, Crawley, WA 6009, Australia; 3 Ruah Community Services, 255 Hay Street, Subiaco, WA 6008, Australia; Ellie.Tighe@ruah.org.au (E.T.); margaret.potangaroa@ruah.org.au (M.P.); leah.watkins@ruah.org.au (L.W.)

**Keywords:** homelessness, indigenous homelessness, kinship obligations, Australia, Housing First, Aboriginal homelessness, family-centred, participatory action research

## Abstract

Background: Aboriginal Australians are disproportionately affected by homelessness, with traditional housing models failing to recognise the importance of kinship obligations and ongoing systemic racism. The Wongee Mia project is a pilot initiative emerging out of a Housing First project tackling homelessness among Perth’s most vulnerable rough sleepers. The project takes a different approach to working with and providing long-term housing to Aboriginal families in Perth, Western Australia. Methods: The Wongee Mia project is centred around one person “Robby” and his family to prevent eviction. Data are collected from monthly action research meetings, yarning sessions with family Elders, and case notes. Results: The project identified 32 family members who had potential to place “Robby’s” tenancy at risk. As at December 2019, 29 members of Robby’s family have been supported by the Wongee Mia case workers, and five have been housed. Key elements of Wongee Mia are the broader links to end homelessness initiatives (the Housing First program), the cultural backgrounds of the case workers and their ability to connect in a meaningful way with the family, Elder involvement (including the co-production of this paper), and an underlying action research model enabling program delivery improvements. Conclusion: The Wongee Mia project offers an innovative way of working with families to prevent unnecessary eviction by working through the whole family’s needs rather than those of an individual in relation to housing.

“Unlike the common colonialist definition of homelessness, Indigenous homelessness is not defined as lacking a structure of habitation; rather, it is more fully described and understood through a composite lens of Indigenous worldviews. These include the following: individuals, families, and communities isolated from their relationships to land, water, place, family, kin, each other, animals, cultures, languages, and identities. Importantly, Indigenous people experiencing these kinds of homelessness cannot culturally, spiritually, emotionally, or physically reconnect with their indigeneity or lost relationships.”

—Dr. Jesse Thistle, 2017 [1]

## 1. Introduction

This paper is about an initiative emerging out of Perth, Western Australia (WA), which is taking a different approach to addressing Aboriginal homelessness. The Wongee Mia project is a pilot initiative emerging out of the 50 Lives 50 Homes project, a Housing First collective impact project tackling homelessness among Perth’s most vulnerable rough sleepers. Housing First is an approach to working with people experiencing homelessness that prioritises safe and permanent housing as the foundation to addressing peoples’ health and social needs [2,3].

This paper has been co-produced by Aboriginal Elders, Wongee Mia case workers, service managers, and the evaluation team. It is the product of a series of yarning sessions, writing workshops, and action research meetings discussing the Wongee Mia model and the challenges experienced by homeless Aboriginal and Torres Strait Islander Australians. We acknowledge that while not all the authors were involved in the direct-writing stages of this paper, the ideas, concepts and content included have been co-produced by all of the authors listed.

The paper is structured as follows: first the challenge of Aboriginal homelessness and the struggles many Aboriginal Australians face accessing affordable and appropriate housing and sustaining tenancies is highlighted. Then the Wongee Mia approach is explained, outlining the program’s critical success factors. Finally, some of the challenges and limitations experienced by the Wongee Mia project in the context of the wider challenges facing Aboriginal Australians who are experiencing homelessness are discussed.

### 1.1. Aboriginal and Torres Strait Islander Homelessness in Australia

The rate of Aboriginal and Torres Strait Islander homelessness in Australia is substantially higher compared with that of their non-Aboriginal counterparts, with Aboriginal and Torres Strait Islanders 10 times more likely to experience homelessness [4].

The colonisation of Australia, and the consequential racialised government policies have carved up and bounded urban communities, driving Aboriginal and Torres Strait Islander peoples out of traditional lands and away from culturally significant sites [5]. In Western Australia, one such policy was the Aborigines Act 1905 (WA) [6], which granted police extensive powers of surveillance and enforced segregation [7]. From the late nineteen twenties until the mid-nineteen fifties, it was illegal for a Noongar person (a person of the south-west of Western Australia) to come into the Perth “town” centre [7].

Dispossession from land and culture remain significant contributors to Aboriginal homelessness in Australia today [8,9], with the legacy of assimilation policies intensifying homelessness through intergenerational trauma, cultural oppression, racism, poverty, lower education, and unemployment [1,9,10,11]. People experiencing homelessness often have a history of adverse life experiences and trauma [12,13]; specifically for Indigenous peoples, trauma and the sense of “not belonging” are intrinsic consequences of the lasting impact of colonisation and contribute to the disproportionate rates of Indigenous homelessness [14,15,16]. Kinship and cultural obligations to family members, distrust of mainstream institutions, overcrowding, and different cultural understandings between Indigenous and non-Indigenous people (such as the definition of homelessness prefacing this article) further exacerbate these disparities [1,17,18,19,20].

Aboriginal and Torres Strait Islander families are over three times more likely to experience overcrowding [21] and, as recognised in the Australian Census definition of homelessness, overcrowding is often a marker of homelessness. It is important to note however, that the very concept of overcrowding, and what “defines it” is not an Indigenous construct and does not do justice to Aboriginal notions of familial and cultural responsibility to take in people without a safe place to stay [21]. Nonetheless, overcrowding can contribute indirectly to the risks of tenancy eviction and homelessness, as overcrowding has been associated with family and domestic violence, damage to property, noise, and antisocial behaviour, which contribute to loss of tenancy and homelessness [20]. As observed in the 50 Lives 50 Homes project, if there are cumulative evictions within a family group these can create a “domino effect” further amplifying the aforementioned risk factors. These additional challenges can put Aboriginal and Torres Strait Islander peoples at greater risk of returning to homelessness [8,22,23].

Strong kinship relations and pressures to accommodate extended family members can make the search for appropriate housing for homeless Aboriginal and Torres Strait Islander families challenging [23]. This is made more difficult by the absence of adequate investment in social housing to meet demand [24]. While homelessness among Aboriginal and Torres Strait Islander families can lead to overcrowding, there is a dearth of appropriate housing for large and extended families. “Official” measures on overcrowding reported through the Australian Bureau of Statistics are likely to significantly underestimate overcrowding as there is a degree of caution about disclosing who lives at a property for fear of consequence [22].

Therefore, demand for appropriate and affordable housing options for Aboriginal and Torres Strait Islander peoples grossly outweighs supply, with implications for Housing First programs and services working with homeless Aboriginal and Torres Strait Islander peoples [3,25].

There have been calls for different approaches to addressing Aboriginal and Torres Strait Islander housing for decades [26], but there have been few examples on how to practically implement these.

### 1.2. Wongee Mia: A Different Approach

Wongee Mia is a special initiative specifically designed to house and support Aboriginal people (from a Noongar community) who are chronic rough sleepers in Perth. Initially called the “Aboriginal Housing Initiative” it was developed from small seed funding in response to a recognised gap of the 50 Lives 50 Homes program [3]. The initiative was developed in consultation with both Indigenous staff within Ruah Community Services (the backbone organisation for 50 Lives 50 Homes) and with specialist Aboriginal organisations. The project developed strong ties with the family Elders and as a result the project was renamed Wongee Mia. The family named the project after the grandmother of the central family member, who was an advocate for strengthening family ties and providing shelter, her family call her “Wongee” meaning strong woman, with “Mia” meaning home in the Noongar language.

“Thinking-outside-the-box” approaches are needed that are driven by and responsive to the needs of Aboriginal people themselves. The Wongee Mia project supports chronic rough sleeping Aboriginal people via the input of Elders on relationships, preferred living arrangements, and culturally appropriate responses. In contrast to traditional one-to-one case management models, the Wongee Mia project takes a “family-centred approach”, whereby the total caseload is the whole family of one person.

## 2. Methods

This paper discusses the critical success factors of the Wongee Mia model and provides a detailed case study of the family and central family member. Embedded in the Wongee Mia project is an action research methodology that sets the framework for continuous learning. Rather than a static, outcomes-based evaluation, the action research component allows the project to continually respond, reflect, and adapt to challenges and learnings as they emerge.

### 2.1. Participant Recruitment

The Wongee Mia model took a snowball sampling [27] approach to recruitment. The key participant “Robby” was purposively recruited for support, and then all family members that posed risk to his tenancy were approached for involvement in the project. Robby was known to the case worker prior to Wongee Mia commencement. In total, as at December 2019, 29 of Robby’s family including aunties, uncles, cousins, and immediate brothers and sisters have been supported by the Wongee Mia case worker.

### 2.2. Data Sources

Data is collected from monthly action research meetings (12 meetings between May 2018–December 2019), 28 yarning sessions with Elders (May 2018–December 2019), and the Wongee Mia case notes reported in Ruah’s electric client management system [28].

**Figure 1:**
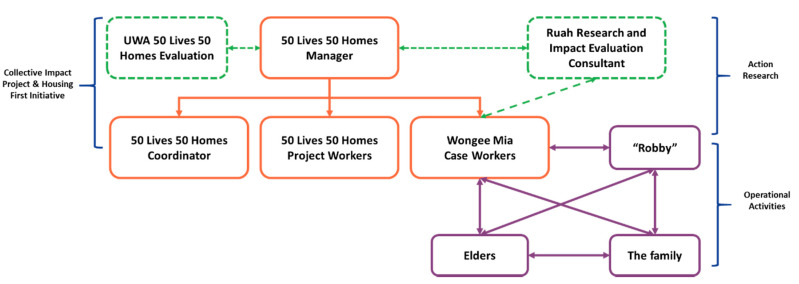
The 50 Lives 50 Homes and Wongee Mia governance structure.

The action research model has two components: (1) participatory action research with Robby’s family. This is facilitated by Wongee Mia case workers through informal yarning sessions with members of Robby’s family, including family Elders, and (2) the project action research team consisting of the Wongee Mia case worker, the 50 Lives 50 Homes manager, and the Ruah Research and Impact Evaluation Consultant (See Figure 1). In May 2019, the project expanded to include one more case worker to support the family. The project team meets monthly to reflect on the project and learnings.

As part of the overarching 50 Lives 50 Homes program, all people completed the Vulnerability Index—Service Prioritisation Index Decision Assistance Tool (VI-SPDAT) to assess for eligibility and to determine risk and vulnerability [29]. The VI-SPDAT vulnerability score draws from the number of years a person has been homeless and the prevalence of risk factors such as substance abuse disorders, mental health issues, untreated chronic health conditions, and poor health behaviours, such as smoking. Self-report data from *n* = 23 VI-SPDATs undertaken between 2014 and 2019 have been presented here and include background information on overall vulnerability and history of homelessness.

### 2.3. Ethics Approval

Approval to conduct the evaluation of the overarching 50 Lives 50 Homes project was granted by the University of Western Australia Human Research Ethics Committee on 20 January 2017 (RA/4/1/8813).

## 3. Results

### 3.1. Case Study

The Wongee Mia program works with one individual “Robby” and the family members that place his tenancy at risk, to prevent him from being evicted.

#### 3.1.1. About Robby

Robby is an Aboriginal male in his late thirties, who first experienced homelessness during mid-adolescence. Robby is part of an extended Aboriginal family with connections to land in both regional and metropolitan WA. Throughout his childhood and formative years Robby was exposed to extensive inter-generational trauma, family violence and “Sorry Business” (Sorry Business is a term used to describe a broad range of practices around death, dying, and funerals by Aboriginal Australians [30]).

Since 2012, Robby has had multiple social housing placements, all of which he lost due to issues of antisocial behaviour, overcrowding, and property damage.

The Wongee Mia project begun supporting Robby in May 2018. Prior to Wongee Mia, Robby was supported through Street-to-Home, which is a homeless outreach program [31] and the 50 Lives 50 Homes Housing First program [3]. The Street-to-Home project enabled Robby to access a tenancy in November 2017.

Robby has successfully sustained his current tenancy since November 2017 through the support of the 50 Lives 50 Homes program and more directly through the Wongee Mia project.

#### 3.1.2. About Robby’s Family

Within the first couple of months of the Wongee Mia project, the Wongee Mia case worker, evaluation consultant, and Robby mapped his family “Yarning Tree”. This identified 32 family members (with 15 different family names), of which 18 were currently homeless rough sleepers. Seven family members were placing Robby’s tenancy at immediate risk, and a further 18 were occasional overnight visitors (*n* = 9), other street-present family members (*n* = 2), or in housing and potentially needing support in the future (*n* = 7) (Table 1).

The 50 Lives 50 Homes project uses OrgCode’s VI-SPDAT to triage individuals most in need of long-term supports and stable housing. Data from 23 individual VI-SPDATs were available for analysis (Table 2). The majority (74%) of responders scored ≥10, indicating they are highly vulnerable and need to be prioritised for permanent housing alongside wrap-around supports, such as that advocated for through the Housing First model [32,33]. In addition to Robby’s long history of homelessness, his family also had extensive homelessness histories, with an average of seven years spent homeless prior to completing the VI-SPDAT (note this could under represent total time homeless if they completed the survey in 2014 and remained homeless for the following six years). While not reported in this paper, the majority of family who completed the VI-SPDAT had multiple physical and mental health concerns, adding a further challenge to being able to rapidly house them.

### 3.2. Key Elements of the Wongee Mia Model

**Figure 2:**
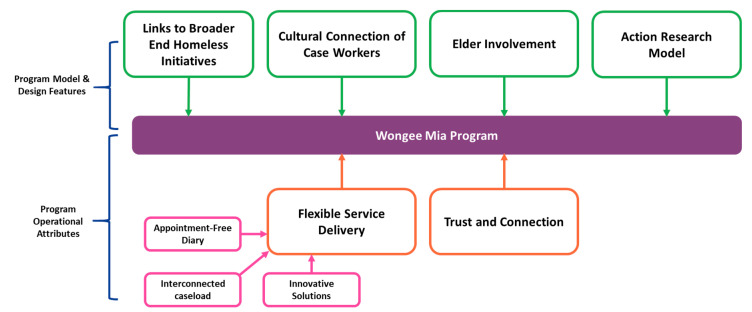
Key elements of the Wongee Mia model.

The Wongee Mia model was inspired by the journey of Robby, his experiences, and the interconnectedness of his experience with that of his family. Six critical factors to the program’s design have been elicited and are described below (Figure 2).

#### 3.2.1. Links to Broader End Homelessness Initiatives

Housing First: The Wongee Mia project is an innovate initiative of the wider 50 Lives 50 Homes Housing First program. The idea for the project was established by the 50 Lives 50 Homes manager, in response to the emerging pattern of Aboriginal 50 Lives 50 Homes program participants returning to homelessness shortly after being housed. As part of the overarching 50 Lives 50 Homes program, strong ties have been established with housing providers (Public Housing, Community Housing, Aboriginal Housing, and Affordable Housing providers).

A key focus of work was working with the housing providers to overcome their concerns that Robby’s family would be “unreliable tenants”—demonstrating to them the successes achieved through protecting Robby’s tenancy. Since May 2018, the Wongee Mia case worker has been engaging homeless members of Robby’s family, signing them up to the Wongee Mia project and working with them to complete applications for public housing. Public housing applications in WA are waitlisted via a general and priority waitlist. There have been 11 housing applications completed since June 2018, both applications for the general housing waiting list (all 11) and additional applications for the priority waiting list (8 of the 11 applications).

Since the project started, four family members have moved into stable long-term accommodation (previously staying in temporary accommodation or rough sleeping), and six are in temporary accommodation (previously rough sleeping). Additionally, Robby has sustained his property for 2.5 years, the longest period he has kept his social housing for.

**Figure 3:**
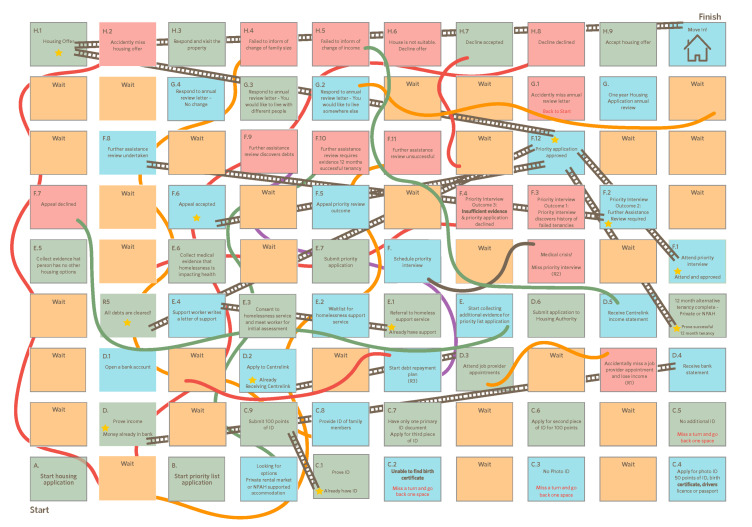
“Snakes and Ladders” to obtain a housing authority property in Western Australia. This diagram has been developed by Ruah’s Wongee Mia Action Research Group (L.W., M.P., E.T.).

Alliance to End Homelessness: Through 50 Lives 50 Homes, the Wongee Mia project is connected to a wider campaign focused on ending homelessness in WA. This has facilitated connection between the activities conducted by the Wongee Mia case management team and family at the grassroots to wider systemic advocacy and campaigning. For instance, in February 2019 the Action Research Team designed a “Snakes and Ladders” of all the required steps required to successfully submit an application to the housing authority (Figure 3).

#### 3.2.2. Flexible Service Delivery

The pilot status of the project and the means by which the project was planned and designed have enabled the Wongee Mia case workers to adopt a flexible support approach.

Appointment-free diary: The Wongee Mia case worker is physically based at a homeless day centre in Perth and the day centre is a known and trusted place to the family. Rather than structured one-to-one appointments, the Wongee Mia case worker has an open diary and family members can visit her at the centre whenever they wish to engage with the service. This provides a sense of control to the family as they can seek support when they require it rather than at set times.

Interconnected caseload: The caseload structure and eligibility are orientated around the central Wongee Mia family and their needs. As most of the family members are currently homeless, the Wongee Mia case worker supports the whole family. Support includes assisting with accessing affordable and appropriate housing, engaging with public housing providers and other Aboriginal and community service organisations (including family and domestic violence support services, health, police and justice, drug and alcohol support services, and financial support services). Supporting the family at the same time through this interconnected caseload enables a more comprehensive approach to preventing Robby from losing his property.

**Figure 4:**
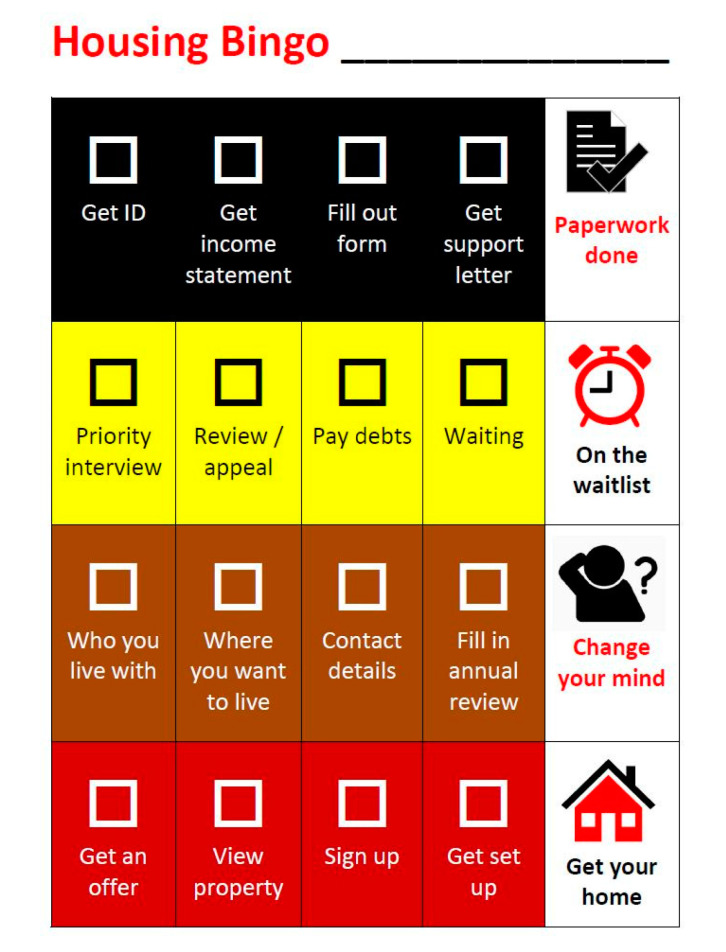
Housing Bingo card. This diagram has been developed by Ruah’s Wongee Mia Action Research Group (L.W., M.P., E.T.).

Innovative solutions: As Wongee Mia is a pilot, there has been a lot of flexibility to trial new approaches. Due to low literacy levels, the Wongee Mia case worker simplified the housing application form and developed a “Housing Bingo” card to make communicating the different stages of the process simpler (Figure 4). The Bingo provides a lay-friendly solution so that each family member can see the current status of their housing application, understand the different administrative layers, and plan what needs to be carried out at each step.

#### 3.2.3. Family Elder Involvement

The Wongee Mia case workers have built strong relationships with Robby’s family Elders. The Elders guide the project in terms of connection to spiritual places and understanding of housing needs and location. This is highlighted in the quote below drawn from one of the yarning sessions with the Elders:

“Over time they lost so much space that have moved into homelessness - You are focusing on housing and they need belonging... I am not an educated man but have experience of my mob and the trauma they have been through... surviving on the streets takes the belonging out... my people are troubled and we need to do more to help them.”

—(Action Research Notes, from Yarning Group with Elders, August 2018)

The Elders connected the difficulties Robby and his family have experienced to the loss of sense of “belonging” experienced as a result of losing land. The Elders have advocated strongly for cultural practices to be included in the project, such as, opportunities to connect back to culture through smoking ceremonies and trips to the bush. They have also shaped the project through sharing their deeper understanding of the importance of connection to home and country and the racial experiences and tensions the Aboriginal community face in accessing appropriate housing.

“We do the good things, but we have to fight to stay in the housing… be prepared for the racism… this pushes people out on to the streets… or be put in the wrong place… it’s not just a focus on housing, it’s the neighbourhood… they need to be close to their own.”

—(Action Research Notes, from Yarning Group with Elders, August 2019)

The Elders have provided guidance on housing location and housing suitability. This has included accompanying the Wongee Mia case worker to meetings with the WA Housing Authority, advocating on the family’s behalf through sharing their own experiences, helping the WA Housing Authority workers understand the family’s unique cultural needs.

#### 3.2.4. Connections and Conversations

A key factor underpinning the project is the strong level of trust between the Wongee Mia case workers and Robby’s family, including the family Elders. The Wongee Mia case worker connected with Robby’s family through conversations about country and family connections, asking “where you from?” and “who’s your mob?”. These connections and conversations were then used as a foundation for understanding the families’ housing and support needs. The case workers have demonstrated their reliability to the family by undertaking tasks and tests set by the family, to prove that they are trustworthy to undertake the more comprehensive task of housing them. The Wongee Mia case worker describes being consistent, taking the time each day to sit and listen to demonstrate that they could be reliable and trusted have been key in establishing a relationship with the family. Other family members have seen this relationship developing and strengthening overtime, so they have then also felt they could trust and work with the Wongee Mia worker.

The trusting relationships that have been established have enabled the case workers to advocate for the family in a number of circumstances such as advocating on Robby’s behalf at the Housing Authorities’ Disruptive Behaviour meetings [34]. Between 2018 and 2019, Robby received three “Strikes” for anti-social behaviour due to family members staying in his house, and the case worker was able to act as his representative in these circumstances and provide evidence to prevent him from being evicted.

There have been instances throughout the project (such as illustrated in Figure 3) where bad news has to be broken to people (delays to applications, wait times for housing, strikes, or eviction notices, etc.), and this, of course, has not been without its challenges. However, both case workers have taken considerable time to make genuine and authentic relationships with the family, and it is through this mutual respect that difficult conversations can be facilitated. The Bingo Card in Figure 4 has also been crucial in managing expectations and providing a visual aid to show the housing process and as outlined in Section 3.2.2, and the open diary enables the family to raise any concerns as they arise.

#### 3.2.5. Case Workers’ Cultural Connections

The Wongee Mia case workers are both Māori and bring their own knowledge of Indigenous ways of doing and understanding of cultural protocols. Case worker 1 is of Tanui/Ngapuhi descent and case worker 2 is of Ngapuhi descent and both have strong connections to culture, which are reflected in their ways of working with the mob. The workers often share their own language and culture with the Wongee Mia family, which has reinforced their relationship and enabled the family to reciprocate and share their own culture back. Within the action research meetings, the Māori concept of “Whakawhanaungatanga” [35], or “sharing time” has been reflected on, as a space for yarning, sharing stories, and sharing meals, and how this has been important to build understandings between each other and informally weaving challenging discussion topics into the sharing space. Sharing common stories of culture, language, and traditions has enabled their acceptance into “the mob”, and the authenticity with which this has been done (with no hidden agendas) has been critical to establishing and maintaining the trust and respect of the family.

The yarning groups facilitated by the case workers are semi-structured in the sense they have an overarching purpose for the day’s yarn and then are opened up to the mob to discuss other topics. The case workers use their cultural connections to support these conversations and use imagery and visual cues to reduce intimidation and pressure for people to participate and not feel shamed if they have not completed their applications.

#### 3.2.6. Action Research

A participatory action research framework is embedded within the project design (Figure 1). The action research team meet monthly to review and reflect over activities and changes from the past months and plan for the forthcoming months. The reflections take the structure of discussing the month’s actions and activities undertaken by the Wongee Mia case worker, reflecting on their meaning and connection to previous knowledge and experiences. The notes are recorded in a cyclic format under the headings “Observation, Reflection, Plan, Action” [36].

The concept of sharing time is embedded into the way the action research is carried out. The monthly action research sessions are a free space for the Wongee Mia team to reflect on the project and topics of significance discussed during the yarning sessions. For example, the action research meetings have explored the complexity of the public housing system that homeless Aboriginal Australians are forced navigate, the challenges and experiences of systemic racism in the private rental sector, and strategies to connect and learn from Aboriginal Community Controlled Organisations who also provide supports to the Wongee Mia family. These sessions have been responsible for producing new ideas and approaches, such as the Wongee Mia Housing Bingo (Figure 4), Snakes and Ladders (Figure 3), and yarning board.

These sessions are facilitated by the 50 Lives 50 Homes project manager and structured around three action research questions (Table 3). Discussions from the action research meetings flow into the yarning groups with Robby’s family and the case management work carried out by the Wongee Mia case worker (Figure 1). The relevance of the action research questions was reviewed and the revised at the end of the first year of the project.

## 4. Discussion

The Closing the Gap campaign has been one of the most prominent federal policy initiatives in Australia. However, despite known links between access to stable housing and the social determinants of health, ensuring affordable and appropriately sized housing for Aboriginal and Torres Strait Islander peoples was not included among the original seven priority targets [37]. With so many Aboriginal Australians experiencing homelessness, it is no wonder that five of the seven targets relating to school attendance, life expectancy, educational achievement, and employment are not on track. Without a safe and secure place to call home, school attendance, employment, and health become inconsequential in the hierarchy of needs [38].

While the Wongee Mia project is small in nature (only working with one family), it is an example of an innovative approach that is using self-determination to address Aboriginal homelessness. In addition to self-determination being a fundamental freedom as outlined in the United Nations Declaration of the Rights of Indigenous Peoples (UNDRIP) [39], the specific need for self-determination in Aboriginal housing responses was recently highlighted by the Victorian Aboriginal Housing and Homelessness Framework for housing responses to be designed “for” and delivered “by” Aboriginal peoples [40]. While the key aim of the framework is to increase the number of Aboriginal homeowners, programs such as Wongee Mia provide a footing for individuals to choose how they want to exit homelessness (whether it be via social housing, private rental, or indeed homeownership). The scaling up of programs such as Wongee Mia to not only to help address the disproportionate rate of homelessness experienced by Aboriginal peoples in Australia [4] but also to simultaneously educate the housing providers of differing cultural needs and obligations to prevent high rates of unnecessary eviction. It has been estimated that an eviction in WA costs over $10,000 AUD [41], so every eviction avoided due to support provided means there is a potential fiscal incentive to keep people in their homes.

The Wongee Mia project is a pilot that grew out of grassroots discussions about how Aboriginal participants in the 50 Lives 50 Homes project can be better supported and to respond to the higher rates of tenancy loss among Aboriginal participants rather than through a top-down government directive. Other studies and attempts to indigenise the Housing First model, such as Distasio et al.’s 2019 [42] analysis of the “At Home Chez Soi” (AHCS) project in Canada, and the AHCS project more broadly, have focused on the principles and governance of indigenising the Housing First model. For instance, focusing on the distinct phases of program delivery and broad community engagement at the national level. Our paper contributes to this work through a close examination of a different approach emerging directly from the community.

A key success has been the relationship established between the project and the family Elders. Often within projects of this nature, connection to Elders is sought at the community level. Whilst such Elders provide valuable community and cultural understanding, this project was able to go deeper through building relationships with Elders in the family the project was seeking to help. A key recommendation arising from a Department of Families, Housing, Community Services, and Indigenous Affairs (FaHCSIA) report on why special services are needed to address Indigenous homelessness was that housing managers and town authorities should work alongside Aboriginal leaders to address homelessness issues [43]. The Wongee Mia project is an example of how successful collaboration between community service providers and Elders can be achieved. Indeed, a critical strength of the project (and the development of this paper) is the insights and historical experience shared by the Wongee Mia Elder, who is also a co-author on this paper.

Finally, we consider the action research component of the Wongee Mia project a flexible and culturally appropriate means to monitor and evaluate the project outcomes. This offers an option to close the gap identified by Distasio et al., 2019, who identify that existing research and assessment tools lack cultural appropriateness and that building meaningful relationships is difficult to count [42]. The action research meetings were cultivated as an organic space for reflection on the project outcomes and learnings generated. However, no family members or project Elders sat in these action research meetings.

### Limitations

As a pilot project, the Wongee Mia model was developed concurrently with its implementation. While resource intensive, we had flexible funding to enable this iterative approach, but this limits potential replicability. Time taken to build relationships, yarn with family members, and understand their experiences does not fit neatly into clinical interventions, which are the mainstream of health funding. In addition, the project and experiences described here are of one Noongar family and, thus, the learnings need to be considered in this context; the generalisability and applicability to expand to other Aboriginal, Torres Strait Islander, or Indigenous populations may not be appropriate. To scale the project and develop as an approach to ending Aboriginal homelessness or addressing other Aboriginal health issues, such as Aboriginal social and emotional well-being, requires long-term commitment to funding resource-intensive models and developing different ways of working.

While the project was designed to proactively find permanent housing for the rest of Robby’s family, housing providers did not always understand this dimension of the projects aims. A key challenge to the success of the Wongee Mia project was the systemic shortage of affordable and appropriate housing and the complex hurdles all vulnerable people are forced to jump through in order to access public housing. Currently in Western Australia, there is up to a nine year wait for public housing [3] meaning that there have been limited people housed via this project and limited options surrounding the types (design and functionality) and location of housing they could access. To date, while only five individuals (including Robby) are permanently housed, all remain in their housing, and substantial time and effort was spent completing the necessary paperwork to join the 50 Lives 50 Homes project and apply for housing, for instance, service consents, application forms, sourcing missing ID, completing VI-SPDAT, reviewing finances, etc. The private housing market is no more accessible with an absence of housing affordability and high levels of competition to access the more affordable units [44]. This has also been identified in a study by Anderson et al., 2016, who also draw attention to the discrimination that Aboriginal peoples experience from landlords and real estate agents, despite submitting numerous unsuccessful applications [22]. These difficulties applying and accessing appropriate housing are not unique to the Wongee Mia, or Perth, context [42]. This is made more difficult in the Aboriginal context with the existence of systemic racism and the fundamental importance of connection and belonging within any tenancy.

## 5. Conclusions

Aboriginal and Torres Strait Islander peoples remain disproportionately affected by homelessness compared to their non-Aboriginal counterparts, with the evidence indicating that there is an urgent need for culturally sensitive approaches to ending Indigenous homelessness. The Wongee Mia project provides one example of how to Indigenise the Housing First model to house whole families in a culturally sensitive and respectful manner. While the program is in its infancy, it provides a culturally safe and appropriately tailored framework that is centred around the family and their needs, to create and foster safe environments that enable Aboriginal and Torres Strait Islander peoples to thrive. Culturally tailored programs consistent with UNDRIP that are centred on self-determination and trust, such as the Wongee Mia model, should be rolled out to other communities (and adapted to the local community context) to determine whether this is a viable model to end Indigenous homelessness.

## Figures and Tables

**Table 1:** Family members placing tenancy at risk.

Risk Placed	*n*
Street-present family member, regular overnight visitor (high risk)	7
Street-present family member, occasional overnight visitor (medium risk)	9
Other street-present family member, not likely to be an overnight visitor (low risk)	2
Non-homeless family member, may require future support (no current risk)	7
Non-homeless family member, not likely to need support (no risk)	7
Total	32

**Table 2:** VI-SPDAT domains for individual Wongee Mia family members.

Domain	*N* = 23
VI-SPDAT Score	
Average Score	10.7
Range	3–16
VI-SPDAT Score ≥10 ^^^ (%)	74%
**Time Spent Homeless Prior to Survey**	**Years**
Average length spent homeless	7
Range	0.7–30
Spent longer than 5 years homeless (%)	52%

^^^ Note a score ≥10 indicates high vulnerability.

**Table 3:** Yearly research action questions.

Year 1 Action Research Questions	Year 2 Action Research Questions
(1) What does it take to work out how to support Aboriginal peoples as an interconnected family system, not just individuals? (2) What does it take to work with housing providers to develop new housing models? (3) What does it take to build solid understanding of best-practice research in this area?	(1) What does it take to increase housing access for Aboriginal families? (2) What does it take to provide Aboriginal families the support needed to access and maintain housing? (3) What are the underlying cultural practices and learnings that have emerged while addressing (1) and (2)?
